# Market trends and ethnobotany of orchids of Mount Cameroon

**DOI:** 10.1186/s13002-019-0308-1

**Published:** 2019-06-25

**Authors:** B. A. Fonge, S. E. Essomo, T. E. Bechem, P. T. Tabot, B. D. Arrey, Y. Afanga, E. M. Assoua

**Affiliations:** 1 Department of Botany and Plant Physiology, Faculty of Science, University of Buea, P.O. Box 63, Buea, Cameroon; 2 Department of Agriculture, Higher Technical Teachers’ Training College Kumba, University of Buea, P.O Box 63, Buea, Cameroon; 3 Douala, Cameroon

## Abstract

**Background:**

The Orchidaceae are noted for their floral diversity. In the Mount Cameroon Region (MCR), the genus Bulbophyllum is the most represented genus of the entire flora of this region. Despite the large number of different species in Orchidaceae, very little is known and documented about the orchids in Cameroon at large, in the MCR in particular. Orchids are widely used in flower gardens and trade. The aim of this study was to assess the floristic value of the orchids compared with those of other parts of the world and equally assess species which are used in herbal medicines.

**Methods:**

This research was carried out in the MCR and the main flower market in Cameroon. Semi-structured questionnaires were administered to vendors, buyers, and cultivators at a ratio of 1:1:1, as well as to herbalists and cultural use indices computed. A total of 107 flower gardens in all gardening localities (07) of the region were sampled.

**Results:**

A total of 66 out of 107 gardens had at least one orchid species. Five orchids which are hybrids cultivated by vegetative propagation are traded as ornamental plants in the MCR. A total of 23 species were used for herbal treatment of certain ailments. The ethnobotanical richness of orchids was scored at 6.86. *Ansellia africana* had the highest cultural importance index (CI), relative importance index (RI), and the relative use index (RNU) while tradactyle tridactylites had the lowest. It was also found that most orchids were used for clairvoyance that is highly associated with myths or folklore and also for external application.

**Conclusion:**

The level of awareness on uses of orchids in the MCR is low. Many people consume orchids mixed with different species of plants for herbal treatment. Orchid trade is used as a part of subsistence by orchid cultivators and vendors.

## Introduction

While large populations of orchid are still found in their natural habitat, in many parts of the world, their number is decreasing due to high demand and population pressure. Many orchid species are threatened due to habitat destruction and indiscriminate collection [1]. At present, the orchids figure prominently in the Red Data Book prepared by the International Union for Conservation of Nature (IUCN), and the entire family was included in Appendix-II of the Convention on International Trade in Endangered Species of Wild Fauna and Flora (CITES) in 2017, where the international trade is regulated [1].

Orchids are nature’s extravagant group of flowering plants, distributed throughout the world from tropics to high alpine regions. They exhibit an incredible range of diversity in shape, size, and color of their flowers. They are important esthetically and medicinally and are also regarded as ecological indicators [2]. Many orchid species are cultivated for their various economic uses, especially in floriculture. Orchids are grown primarily as ornamentals and are valued as cut flowers because of their exotic beauty, and for most species, the flowers persist for long [3]. Some orchids are used as herbal medicines, food, and other cultural values by many different cultures and tribes in the different parts of the world [4].

Orchids are one of the largest and most diverse groups of angiosperms consisting of nearly 25,000 species with more than 850 genera [5, 6]. The Orchidaceae are the second most diverse plant family in the Mount Cameroon Region after Asteraceae [7]. Some orchids like *Eulophia campestris*, *Orchis latifolia*, *and Vanda roxburgii* have drawn the attention of the scientific community because of their medicinal uses [8, 9]. Medicinal orchids mainly belong to the genera: *Calanthe*, *Coelogyne*, *Cymbidium*, *Cypripedium*, *Dendrobium*, *Ephemerantha*, *Eria*, *Galeola*, *Gastrodia*, *Gymnadenia*, *Habenaria*, *Ludisia*, *Luisia*, *Nevilia*, and *Thunia* [6, 10].

Research has shown that *Dendrobium* spp., *Gastrodium* spp., and the hyacinth orchid (*Bletilla striata*) have been used in the treatment of ailments like hemorrhages of the stomach or lungs, uterine bleeding, and nose bleeds, as well as whooping cough [1], and more medicinal uses are being uncovered across the world [11].

However, in Cameroon, most studies of orchids have been centered mostly on inventory and diversity [12, 13], in spite of the various ways in which Cameroonians use orchids daily, and as a result, very little is known or published about the socio-economic importance and the ethno uses of orchids in Cameroon at large and the MCR in particular. This study therefore aimed at investigating ethnobotany of orchids of the Mount Cameroon Region, in view of bridging this knowledge gap. Specifically, the research addressed the following questions:

1. What orchids are of ornamental importance in the MCR?
2. What orchids have medicinal uses in the MCR and what are these uses?

In horticultural gardens in the MCR and a major market in Douala (Marche de Fleurs), orchids are among the most popular ornamentals traded. We theorized that this orchid trade is fueled by indigenous ornamental and ethnobotanical uses that justify the need for their conservation.

## Materials and methods

### Study sites

**Fig. 1:**
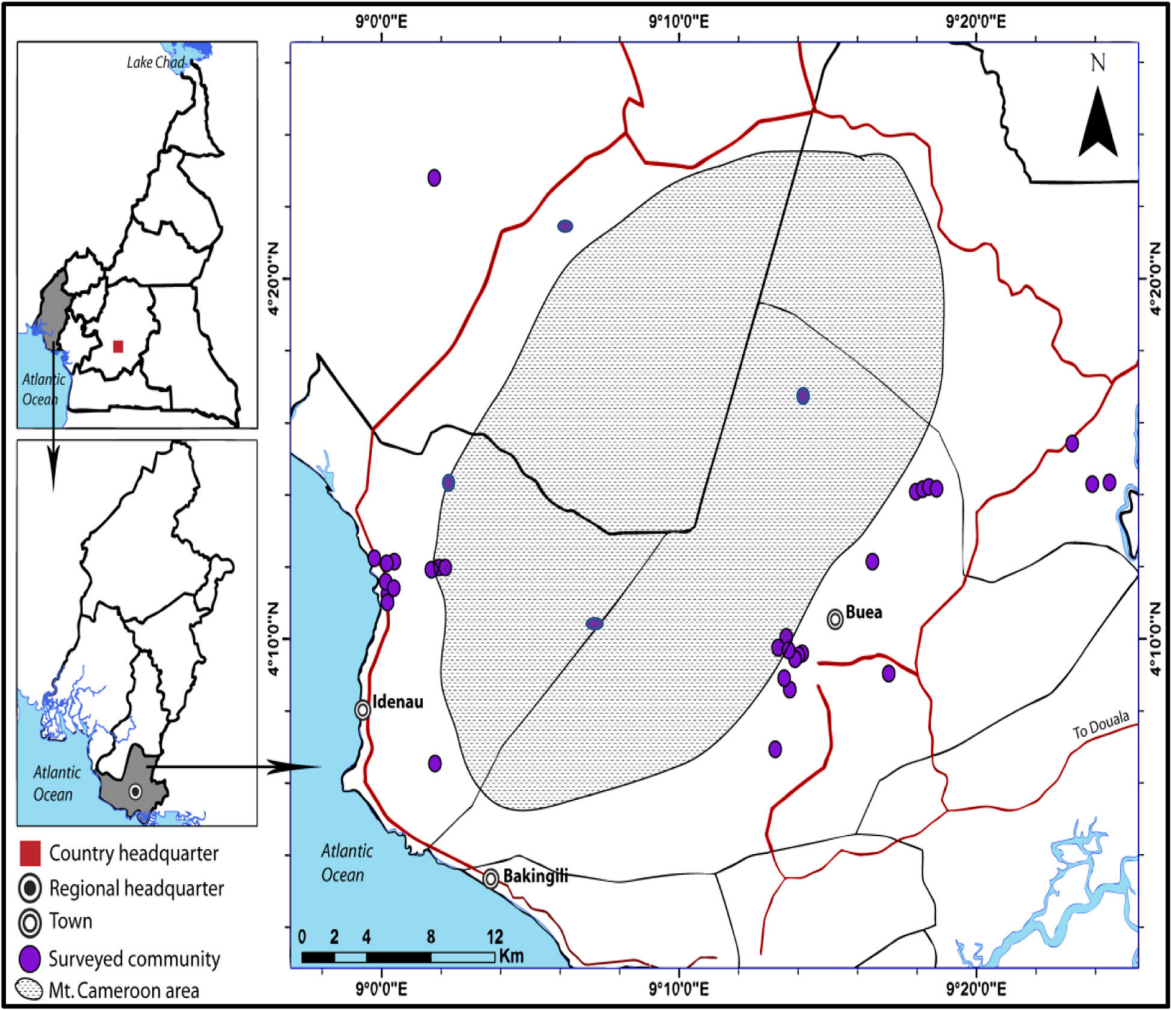
The Mount Cameroon Region

The Mount Cameroon Region as shown in Fig. 1 is situated in the South West Region of Cameroon in Central Africa. The area extends from the Atlantic coast to Mount Cameroon, with an altitude of 4100 m [14]. Mount Cameroon is the highest mountain in West and Central Africa and an active volcano [15, 16]. Due to the volcanic origin, the surrounding soil is rich in nutrients and provides high fertility for both natural vegetation and farmland [17]. The area has a humid tropical climate modified by the topography from sea level to the top of the mountain. The annual rainfall in most of the region ranges from 2500 to 3500 mm, except at Debunscha, which is the wettest place in Africa with a mean annual rainfall of 10,000 mm. The climate of the Mount Cameroon Region is predominantly tropical, with a dry season from November to February and a rainy season from March to October. Rainfall and temperatures diminish and are moderated up the slopes and further inland.

The mean annual temperature is about 25 °C (Fraser PJ, Hall JB, and Healey JR: Climate of the Mount Cameroon Region: long and medium term rainfall, temperature and sunshine data, unpublished). The relative humidity remains at 75 to 80% throughout the year in the south-western side of the mountain, due to marine influence and the incidence of mist and orographic cloud formation.

### Socio-economic survey

This survey was conducted to determine and record the ornamental uses of orchids. It was carried in various locations as follows:

For both surveys, three groups of persons were sampled; these include orchid cultivators, orchid vendors, and buyers. Mixed methods sampling was used in which respondents were identified through snowball sampling methods and data collected through semi-structured questionnaires, combining the rapid rural appraisal (RRA) and the participatory rural appraisal (PRA) approached. A total of 20 questionnaires were administered to each of the following groups: orchid vendors, cultivators, and buyers after obtaining oral Prior Informed Consent, giving a total of 60 interviews. Prior Informed Consent is sought from respondents to a study by explaining the purpose for the study and the personal liabilities of both the researcher and the respondent, and that the respondents can only participate willingly, retaining the right to withdraw from the study at any point is they so choose. This is done prior to each interview. The overall study was approved by the University of Buea Ethics Committee.

For the flower gardens, 107 flower gardens located at Mile 14 Dibanda, Mutengene, Bokwango, Likoko, Bwassa, and Sasse in the MCR were surveyed. The purpose of this survey was to record the type of orchid cultivated and sold as ornamentals.

The main flower market in Cameroon, known by its French appellation as “Le Marché des Fleurs” situated in Douala in the Littoral Region of the country, about 75 km from the study area was also surveyed to record the types of orchids sold and the cost price of respective species.

### Evaluation of medicinal potentials

This study was carried out to discover the possible traditional medicinal values of orchids within the MCR. Herbalists and members of the population who had knowledge about the orchids were surveyed. A snowball sampling technique in tandem with a “show and tell” approach was used, whereby fresh plants and/or good photographs of plants were shown to respondents, followed by the questions about the plants. A total of 50 questionnaires were administered to these two groups of persons after obtaining oral Prior Informed Consent. Voucher specimens of all orchids collected were prepared and taken to the National Herbarium in Yaounde (YA) for identification and preservation.

### Data analysis

The socio-economic values of orchids that determine their floristic or horticultural uses were analyzed using Microsoft Excel 2016 (Microsoft Inc., USA). Ethnobotanical data were categorized into 10 medicinal use categories namely: bone condition, skin conditions, epilepsy, stomach problems, psychological problems, reproductive, pulmonary conditions, clairvoyance, blood-related, and pains. Plants which are believed to be useful as charms with psychic powers were categorized under clairvoyance. These categories were modified from those of [18, 19]. The basic variables like frequency of citation (FC), use reports (UR), number of uses (NU), and ethnobotanical indices such as the relative frequency of citations (RFC), relative importance index (RI), and cultural importance index (CI) were determined using standard procedures as described in [18, 19].

## Results

### Socio-economic survey

#### Distribution of orchids in flower gardens

**Fig. 2:**
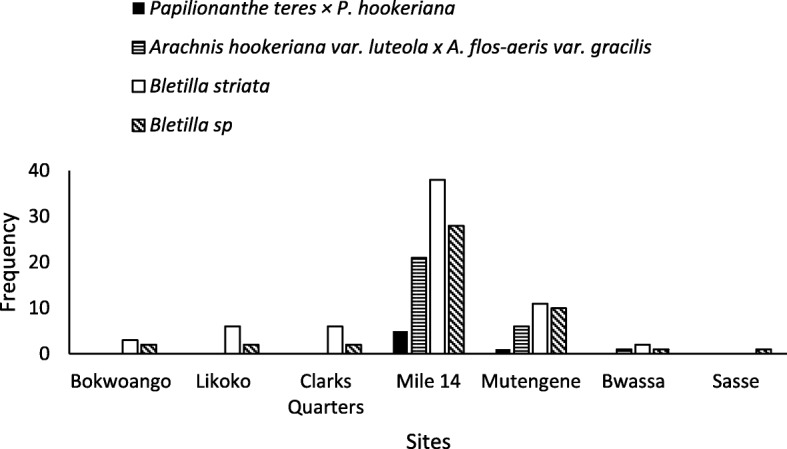
Distribution of orchid species in flower gardens of the MCR

Of the 107 flower gardens that were surveyed in the MCR, 41 gardens did not have any orchid species while 66 gardens had at least one type of orchid species. Only a single garden out of the 5 visited in Sasse had an orchid species (*Bletilla striata*) (Table 1). *Bletilla striata* and *Bletilla* sp. were found in almost all the gardens in Mile 14 Dibanda. Sasse, Bokwango, Likoko, and Clark’s quarters had the least representation of orchid species, while Mile 14 Dibanda had the highest. Of the gardens that had orchids, *Bletilla striata* was present in all (Fig. 2). Mile 16 Bolifamba and Mutengene localities had all 5 orchids species while Bokwango, Likoko, Bwassa, and Clerks Quarter had only two each (*Bletilla striata* and *Bletilla* sp.). The *Arachnis* were found mostly in Mile 14 Dibanda and Mutengene, with a lone stand of the yellow ribbon found in a single garden in Bwassa.

**Table 1:** Common names used in the study area

SN	Scientific name	Common name	Distinguishing names
1	*Papilionanthe teres* × *Papilionanthe hookeriana*	*Vanda* Miss Joaquim	Leafless orchid
2	*Arachnis hookeriana* var. *luteola* × *Arachnis flos-aeris* var. *gracilis*	*Arachnis* Maggie Oei yellow ribbon	Long stem orchid yellow flower
3	*Arachnis hookeriana* var. *luteola* × *Arachnis flos-aeris* var. *gracilis*	*Arachnis* Maggie Oei red ribbon	Long stem orchid red flower
4	*Bletilla striata*	*Bletilla striata*	Orchid pink flower
5	*Bletilla* sp.	*Bletilla* sp.	Orchid purple flower

There were 3 identified species in cultivation in the MCR and one identified to generic level. These included *Papilionanthe teres* × *Papilionanthe hookeriana* (*Vanda* Miss Joaquim), *Arachnis hookeriana* var. *luteola* × *Arachnis flos-aeris* var. *gracilis (Arachnis* Maggie Oei red and yellow ribbon), *Bletilla striata*, and another member of the genus *Bletilla* which could not be further identified in the course of the study. All orchid cultivators used one common name for the different orchids, “Orchidee.” Table 2 indicates the common names used to distinguish the different orchids.

**Table 2:** Distribution of 5 orchids in flower gardens of the different localities of the MCR

Locality	No. of gardens visited	No. of gardens with *Arachnis* Maggie Oei (yellow ribbon)	No. of gardens with *Arachnis* Maggie Oei (red ribbon)	No. of gardens with *Bletilla striata*	No. of gardens with *Bletilla* sp.	No of gardens with *Vanda* Miss Joaquim	No. of gardens without orchids
Bokwaongo	10	–	–	03	02	–	07
Likoko	15	–	–	06	02	–	09
Clarks quarters	10	–	–	06	02	–	04
Mile 14	42	16	05	38	28	05	04
Mutengene	15	04	02	11	10	01	04
Bwassa	10	01	–	02	01	–	09
Sasse	05	–	–	01	–	–	04
Total	107	21	07	67	45	06	41

#### Orchid trade

The trade in orchids in the MCR and the main flower market in Douala is mainly fueled by the cut flowers market (horticultural values), and the prices vary depending on the species.

**Fig. 3:**
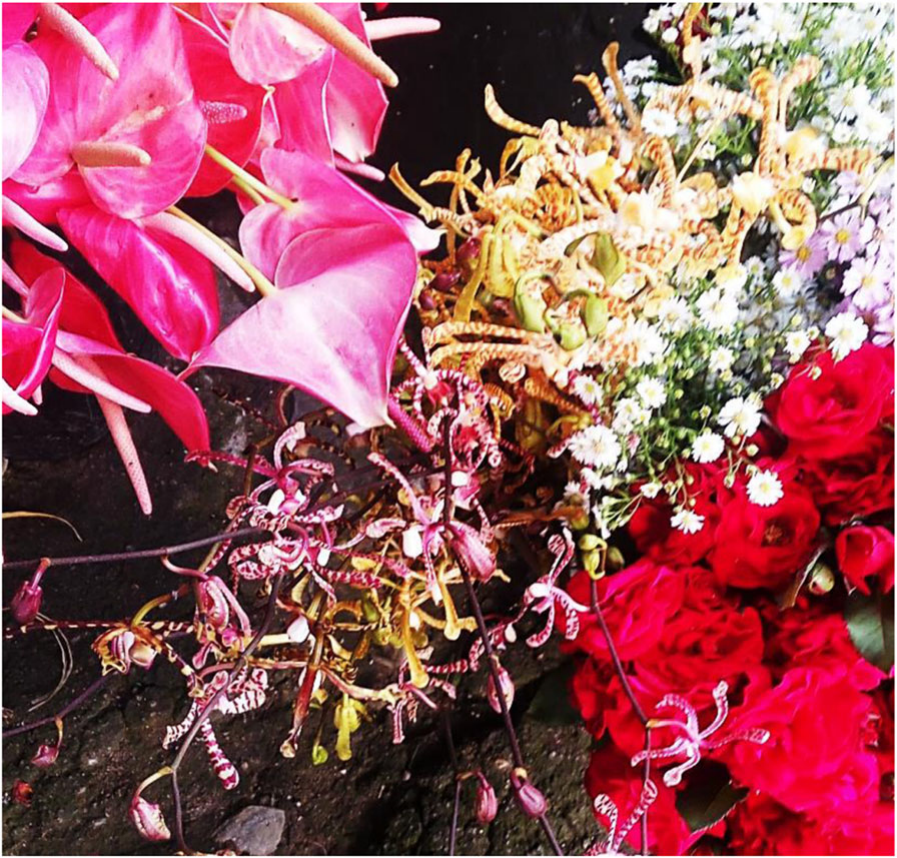
Collection of orchids, roses, and *Anthuriums* sold at the Marché des fleurs in Douala

*Arachnis* Maggi Oei: Its peak marketing period is between December and February during which the prices are much elevated, as well as the months of July and August when the prices fall due to peak flowering periods. A single stalk (inflorescence) of this species is sold at 700 FCFA ($1.25 US) during the dry season and 500 FCFA ($0.9 US) during the rainy season. All *Arachnis* sold in the flower markets were from the MCR. It should be noted that this species is grown by vegetative propagation and was not found in the wild. Florists who indulge its cultivation, from the start of flowering to the end, could have an average sale of approximately 250.000 FCFA ($500 US) per annum. Figure 3 represents a single day quantity of *Arachnis* brought by a flower vendor in the flower market during the month of February.

During this particular day (the weekend before Valentine’s Day), a single inflorescence was sold at 1000 FCFA ($1.7 USD). The total sale at the end of the day was 70.000 FCFA ($125 USD) per trader. It is worthy to note that *Arachnis* could flower as much as twice a year in favorable conditions. When found in a cool place and watered during the dry season, flowering could be induced. The general flowering period is during the rains from the months of July to August. The flowers are very easy to handle and could last from 2 weeks to a month after being cut off from the main plant depending on the storage condition. The red ribbon is often highly demanded than the yellow ribbon. These orchids are mostly mixed with other flowers such as roses and offered as gifts (romantic offers), used in funerals, decorations during marriages, and bouquet for installations and awards ceremonies. In the flower market, orchids are second only to the roses in terms of prices and are always in limited supply.

In the MCR where cultivation takes place, the prices are lower compared to the flower market in Douala. An inflorescence of *Arachnis* is sold at 300 FCFA ($ 0.5 US) to 500 ($ 0.9US) FCFA during the flowering season. Flower pots of the species are prepared and sold at a cost 2.000 FCFA ($3.57 US).

**Fig. 4:**
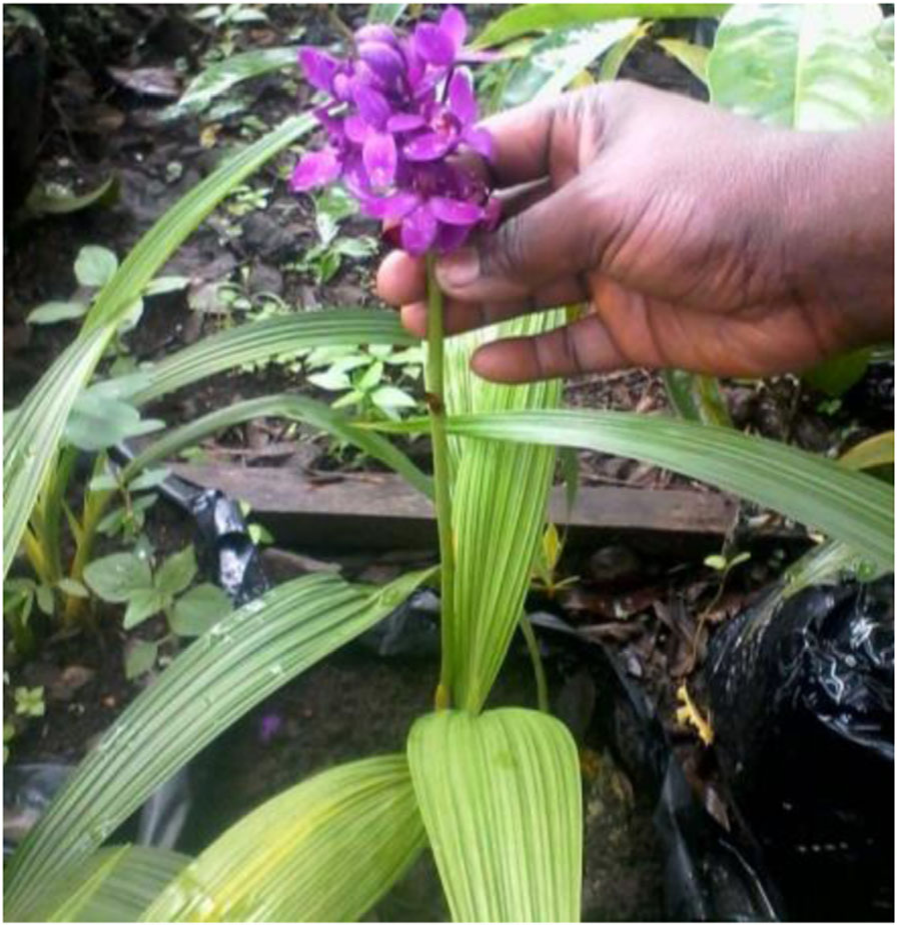
Pot of *Bletilla striata*

*Vanda Miss Joaquim*, *Bletilla striata*, and *Bletilla* sp. are not sold in the flower market in Douala but are of very high demand in the MCR. They are sold as entire potted plants (Fig. 4).

A pot of each species costs from 2.000 FCFA ($3.57 US) to 3.500 FCFA ($ 6.25 US). A florist typically has as many as 100 pots of each species. Most buyers preferred *Bletilla* to *Vanda* Miss Joaquim, planting them as indoor or outdoor ornamentals in homes and hotels. A yearly sale from these potted orchids yields an approximated sum of 550.000 FCFA ($1.339 US) per farmer. These orchid species flower only once a year, and the flowering period of a single plant can last for 2–3 months.

### Ethnobotany of orchids

#### Traditional use of orchids in the MCR

Twenty-three orchids were found to be used in traditional medicine. Some were taken as decoction, concoction, or paste, while others were associated with magic (Table 3). The majority of species used as herbal medicines in the MCR were from the following genera: *Angraecum*, *Ansellia*, *Bulbophyllum*, *Liparis*, *Habenaria*, *Graphorkis*, and *Polystachya*. Traditional healers did however not specify how orchids were prepared for charms. *Ansellia africana* was especially frequently used as herbal medicine. The usage of bulbs was mostly associated with sorcery and for external application. All the orchids used medicinally are obtained only from the wild.

**Table 3:** Orchid species employed in herbal medicines in the MCR

SN	Name of species	Common name	Use	Mode of application	Plant part used	Method of preparation	No of respondents
1	*Ansiella africana* Lindl	Ewomboh	Cough	Concoction	Pseudobulbs	Crush with leaves of *Piper guinensis* boil	11
			Madness	Concoction	Pseudobulbs	In combination with other herbs that were not given is the main ingredient for the treatment of madness	08
						Crush and boil	
			Diabetes	Decoction	Roots	3 leaves, 10 limes, and parasite boiled in 5 L of water and consume every morning	05
					Leaves	Crush with bitter cola (*Garcinia kola*) consume as tea	08
			Impotence	Concoction	Roots	Grind with dried seeds (7) of *Piper guineense* and apply	10
						Macerate leaves and apply before going to bed	
			Side pain	Paste	Leaves	Paste act as short-term contraceptives	15
			Nightmares (bad dreams)	Maceration	Leaves	Not specified	02
			Pregnancy inhibitor	Paste	Pseudobulbs		05
			Love charms	Not specified	Not specified		08
2	*Angraecum augustipetallum* Rendle		Snake repellant	Not specified	Whole plant	Not specified	0
			Bone fortification in children	Maceration	Leaf apex	Macerated leaves with kernel oil (“manyanga”)	12
			Abortion	Concoction	Whole plant	Crush plant and boil	04
3	*Angraecum birrimense* Rolf		Against sorcery	Not specified	Whole plant	Not specified	04
			Snake repellant	Not specified	Whole plant	Not specified	02
4	*Ancistrorhynchus seratus* Summerh	Koh	Diabetes	Tea	Leaves	Dried leaves	06
5	*Bulbophyllum melinostachyum* Schltr*.*		Anti-poisons	Powder	Whole plant	Grind dried leaves to powder and consume	10
6	*Bulbophyllum barbigerum* Lindl*.*		Side pain	Paste	Whole plant	Grind with dried seeds of *Piper guinensis* and apply	12
			Ear pain	Decoction	Leaves	Crush leaves with few drops of lemon or limes (stop mucor). Drop in the infected ear	07
7	*Bulbophyllum intertextum* Lindl.	Mpah	Side pain	Paste (smear)	Whole plant	Grind with dried seeds (7) of *Piper guinensis* and apply	10
8	*Bulbophylum lupulinum* Lindl.	Etumukwoboh	Against sorcery (protection)	Planted (not exposed)	Whole plant	Grown plants treated (sprinkled) once or twice a year with blood of a roster accompanied with spoken desires.	07
			Night poisons	Concoction	Whole plant	Put a bottle of water and placed in the room	03
					Whole plant	Boil with 7 heads of *Ageratum conyzoides* and take as tea	05
9	*Bulbophyllum calyptratum* Kraenzl		Skin diseases (measles, poxes abscesses, rashes)	Maceration	Leaves	Macerate leaves with yellow stone and kernel oil, then apply	12
			Wounds	Decoction	Whole plant	Wash wounds with decoction	06
			Burns	Paste	Whole plant	Apply paste on the burnt spot	10
10	*Bulbophyllum simonii* Summerh		Luck portion	Powder	Whole plant	Mix in body lotion and apply when need be	06
11	*Bulbophyllum falcatum* (Lindl.) Rchb.f.		Against thunder and sorcery	Not specified	Whole plant	Grown in compound	05
			Predictions	Not specified	Leaves	Immediate necrosis young leave means not embarking on a seriously planned journey	07
12	*Bulbophyllum pumilum* (Swartz) Lindl.	Nnokoh	Epilepsy	Concoction	Whole plant	Boil with leaves of *Ageratum cornyzoides* and *Piper guineensis* take as bath and consume as tea	05
13	*Calyptrochilum emarginatum (*Afzel.ex Sw) Schltr.		Charms	Not specified	Leaves	Not specified	15
			Good luck	Power	Leaves	Make power out of *Tapinanthus bangwensis* and orchids, mix with body lotion, and apply	06
14	*Cyrtorchis acuata*		Charms	Powder	Whole plant	Consume as tea	07
			Diabetes	Infusion	Leaves	Dried leaves consume as tea	04
			Skin diseases	Paste	Leaves	Mix a quantity at a time in body lotion and apply	05
15	*Diaphananthe bidens* (Afzel ex Sw.) Schltr.	Mbingwoh	Fertility	Powder	Whole plant	Powder of plant mixed in milk act as an aphrodisiac	03
			Diabetes	Concoction	Whole plant	Boiled mixture of orchid and *Piper umbellatum*	08
16	*Graphorkis laurida* (Sw.) Kuntze	Nkelenkwokwo	Coughs	Concoction	Whole plant	Crush and boil with sweet alligator pepper (*Piper guinenses*) and ginger	13
			Tuberculosis	Powder	Whole plant	Mix powder with black palm kernel oil and consume	09
			Tooth maggot/tooth aches	Concoction	Whole plant	Crush and boil with back of pear	07
17	*Habenaria procera* (Afzel. ex Sw.) Lindl.	Ekuh	Blood purification	Powder	Whole plant	Dried leaves of plants are ground and consumed at such or mixed with lemon and taken as tea	12
			Gastritis (chest and stomach pains)	Concoction	Whole plant	Plants boiled with leaves of *Piper guinensis*	05
			Arthritis	Decoction	Pseudobulbs	Cush, boil and consume. Also massage body with crushed substance	10
18	*Liparis nervosa* (Thunb.) Lindl.		Burns	Paste	Entire plants	Crush and apply to spot	15
			Ulcers	Concoction	Whole plant	Boil with *Bidens pilosa* and leaves of *Piper guineense*, take as tea	08
			Stomach aches	Powder	Whole plant	Consume as tea	10
19	*Listrostachys pertusa* (Lindl.) Rchb.f.		Measles	Concoction	Whole plant	Crush and boil take as purgative	05
			Constipation	Powder	Whole plant	Consume as tea	04
20	*Polystachya concreta* (Jacq.) Garay & H.R.Sweet		Rheumatism Arthritis	Not specified	Not specified	Not specified	01
21	*Polystachya cultriformis* (Thouars) Lindl. ex Speng.	Neshieh	Measles	Concoction	Whole plant	Boil plants with mistletoes (*Tapinanthus bangwensis*) and limes	05
			Burns	Paste	Leaves	Grind leaves with seeds of *Piper guineense* and apply	04
22	*Polystachya caloglossa* Rchb.f.	Ntohpoupou	Rheumatism	Not specified	Not specified	Not specified	02
23	*Tridactyle tridactylites* (Rolfe) Schltr.		Madness	Concoction	Whole plant	Not specified	03

**Fig. 5:**
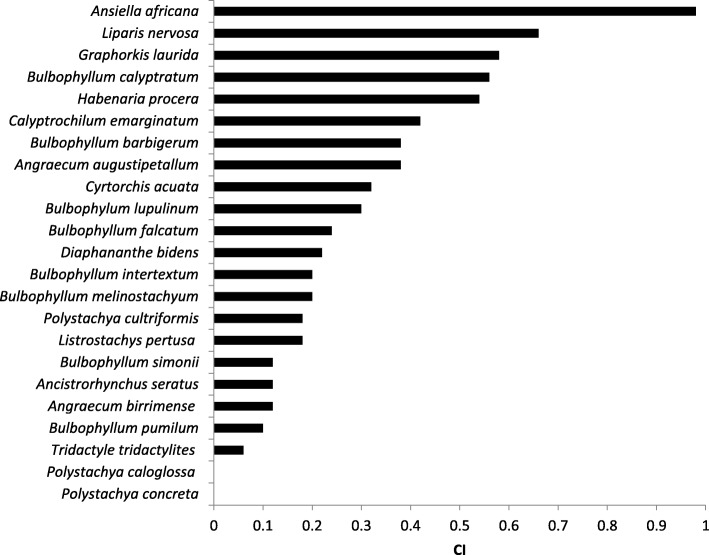
Cultural importance (CI) of orchids used as herbal medicine in the MCR

Species with the highest cultural importance index (CI) were *Ansiella africana*, *Liparis nervosa*, and *Graphorkis laurida* (Fig. 5). Those with the least cultural importance index were *Angraecum birrimense*, *Bulbophyllum pumilum*, and *Tradactyle tridactylites*.

Overall on average, the CI for all orchid species with ethnobotanical uses in the MCR, the ethnobotanical richness, was 6.86. This could be used as a standard for a comparative study of different medicinal plant species of the MCR or could also be used for same species in a different area.

**Fig. 6:**
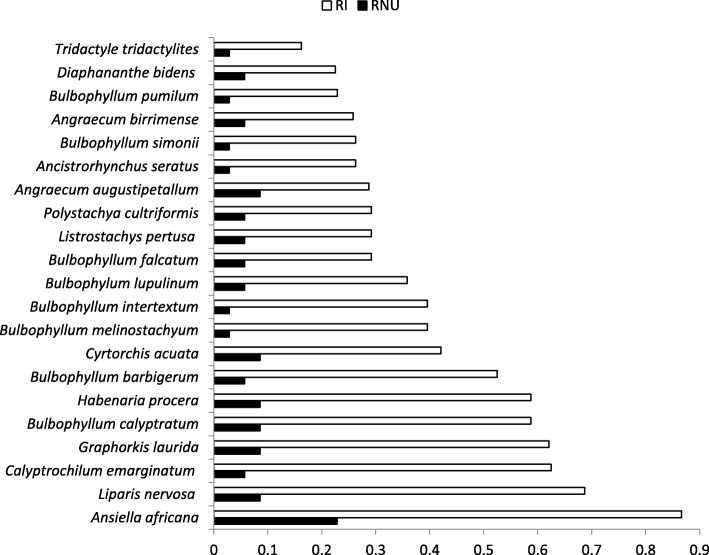
Relative importance and relative use index of orchids employed as herbal medicine

Figure 6 further shows the relative importance index (RI) and the relative use index (RNU) of orchid species. *A. africana* had the highest RI and RNU, while *Chameangis emarginatum*, *B. melinostachyum*, and *B. intertextum* had high RI but very low RNU.

**Fig. 7:**
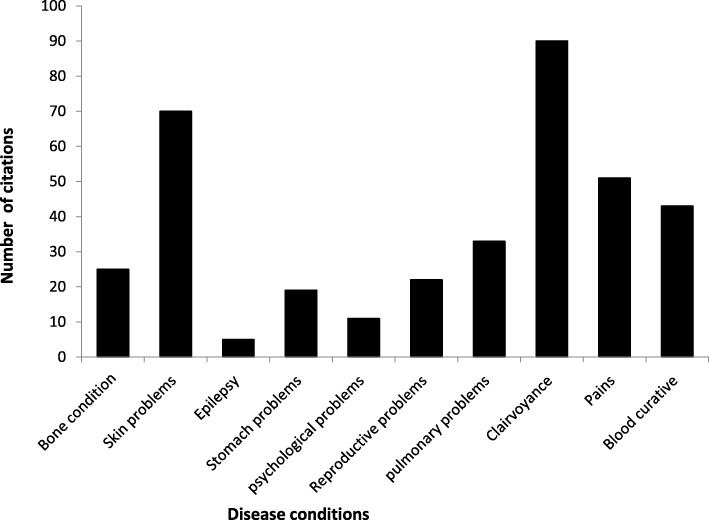
Categorization of the use of orchids as herbal medicine

Most medicinal orchids were used for clairvoyance, highly associated with myths or folklore, and also for external application to cure skin-related diseases as well as for pains (Fig. 7). The least frequent uses were for epilepsy and psychological problems. Only one species (*Bulbophyllum pumilum*) was known to be used as a curative agent for epilepsy.

## Discussion

The purpose of this study was to elucidate the ethnobotanical uses of orchids in the MCR and to complement ongoing efforts for their conservation. Most of the orchids not only had ethnomedicinal uses, esthetic uses, and mystical uses, but also fetched appreciable incomes for traders in orchids as ornamentals. Several authors have found uses for orchids similar to those reported in this study. An example is *Bletilla striata*, an important medicinal orchid, which is reported to have been used in treating wounds in China for over 1500 years [20]; [21]. The pseudobulb is reported to have antibacterial, anti-inflammatory, antiphlogistic, demulcent, styptic, and vulnerary properties [22]; [8]. It is taken internally in the treatment of hemorrhages of the stomach or lungs, uterine bleeding, and nose bleeds and is said to be effective against the endotoxin produced by *Haemophilus pertussis* in whooping coughs.

### International versus national trade of orchids

The rich horticultural values attributed to orchids in the international markets cannot be compared with those of the national flower market in Cameroon, as the “flower culture” is still developing in the country. Many people often prefer synthetic to natural flowers. Although natural flowers are beautiful with a pleasant fragrance, they do have a limited lifespan and cannot last up to a year, unlike synthetic flowers which can last for more than a year. Secondly, natural flowers are comparatively expensive.

Orchids earn a reduced price in Cameroon compared to values obtained in the international markets. A single pot of Vanda Miss Joaquim, *Bletilla striata*, and *Bletilla* sp. in flower gardens of the MCR was sold at 3.000 to 3.500 FCFA (6 to7 $). According to information from Toh Garden [23], Vanda Miss Joaquim was sold at $25 US (approximately 15.000 FCFA) in international trade; *Bletilla striata* at $17US (9.000 FCFA) [24], while the *Arachnis* species were sold on average at $28.95 US [25]. However, orchid traded as ornamentals do have a large local economic impact, and the income is used to buy land, build houses, cover education expenses, and provide for other needs of the orchid-trading families.

### Species with medicinal uses in the MCR

The use of wild species of medicinal orchids seems to be of great economic importance across the developing world. Bulpit [26] reported the use of *Cyrtorchis arcuate* in China; with a similar method of preparation (pounded into a powder) employed in promoting friendship, consistent with its use as a charm in the MCR. It is also reportedly used for treating diabetes and skin infections which is consistent with findings of the current study. Also, *Tridactyle tricuspis* was reported to be used in curing madness [26]; in the MCR, species that were used for the same purpose were *Tridactyle tridactylites* and *Ansiella africana*. Our results are consistent with other studies that found medicinal uses for orchids, for instance, Linthoingambi et al. [27], Subedi et al. [28], and Pant [1] reported the use of the tubers of *Habenaria* for blood purification and the cure for leprosy, unconsciousness, and youthfulness and to increase vigor and *Liparis* sp. for treatment of burns, cancerous ulcers, and gangrene*.*

Dash et al. [29] and Teoh [28] reported the use of *Polystachya concreta* for the cure of arthritis. Similar results were obtained in the present study. Many authors [29–34] found *Ansiella africana* used as charms and antidotes for nightmares, similar to our own results. For such species, the traditional doctors and herbalists interviewed typically do not provide methods of preparation. Love charms have been used extensively in African traditional culture. Some species encountered in this study have similar uses elsewhere, for example, the roots of *A. africana* are prepared as an infusion and administered as an emetic [35], while the leaves of the same species are used by men as a courting charm [36]. This species was reported by Hulmes [37] as a key ingredient in an emetic that is administered by a man to a young lady to make her love him. Species of *Liparis* are used as love or good luck charms though the parts used were not specified [36]. On a specific note, charms are prepared to either prevent or promote fertility*. Ansiella africana* is reportedly used by young men to prevent women from having children if their love is not returned [38]. Protective charms may be used for bad dreams, to ward off evil, to protect one from lightning strikes, and to protect the home. *Ansiella africana* leaves are prepared as an infusion to treat persons experiencing bad dreams [36]. Alternatively, one can inhale the smoke of its burning roots for the same purpose [36]. The tubers of certain orchids are used as an infusion that is sprinkled around the home to ward off evil. Whole plant decoctions of unspecified *Habenaria* species are ingested by couples to ensure the birth of a son [37].

## Conclusion

Three orchid species and another identified to generic levels were of horticultural importance in the MCR. They include *Papilionanthe teres* × *Papilionanthe hookeriana*, *Arachnis hookeriana* var. *luteola* × *Arachnis flos-aeris* var. *gracilis*, *Bletilla striata*, and another member of the genus *Bletilla*. In addition, 23 species of orchids were of ethnomedicinal importance, useful in treating ailments from coughs to various pains. These results are significant as they expose the hidden potentials of orchids of the MCR and provide an added incentive for the conservation of orchids of the region. Sustainable conservation that takes into consideration the ethnobotanic and socioeconomic uses of the species represents a plus-plus approach and would thus be more effective.

## Data Availability

All data relevant to understanding the results of this study have been included in the manuscript.
